# The effects of long-term medication on growth in children and adolescents with ADHD: an observational study of a large cohort of real-life patients

**DOI:** 10.1186/s13034-015-0082-3

**Published:** 2015-10-28

**Authors:** Shelagh Gwendolyn Powell, Morten Frydenberg, Per Hove Thomsen

**Affiliations:** Centre for Child and Adolescent Psychiatry, Aarhus University Hospital, Skovagervej 2, entr. 81, 8240 Risskov, Denmark; Department of Public Health, Aarhus University, Bartholins Allé, build. 1260, 8000 Aarhus C, Denmark

**Keywords:** Central stimulants, ADHD, Growth, Long-term effects

## Abstract

**Background:**

Children and adolescents with ADHD treated with central stimulants (CS) often have growth deficits, but the implications of such treatment for final height and stature remain unclear.

**Methods:**

Weight and height were assessed multiple times in 410 children and adolescents during long-term treatment with CS, which lasted between 0.9 and 16.1 years. Weight and height measures were converted to z-scores based on age- and sex-adjusted population tables.

**Results:**

CS treatment was associated with (1) a relative reduction in body weight and a temporary halt in growth, (2) a weight and height lag after 72 months compared with relative baseline values. No relation to early start of medication (<6 years), gender, comorbid ODD/CD or emotional disorders was observed.

**Conclusions:**

Treatment with central stimulants for ADHD impacts growth in children and adolescents, and growth should be continuously monitored in patients on chronic treatment with these medications.

## Background

The use of central stimulants (CS) for the treatment of ADHD has increased markedly over the past decades [1–4] as has the number of patients remaining in treatment throughout puberty and into adulthood [5, 6]. This development accentuates concern over the long-term side effects of CS treatment in general. The anorexic effect of CS [7–12] and the ensuing initial weight loss [13] is of particular concern for clinicians and parents caring for their children. The long-term impact of CS on growth parameters has therefore attracted much attention both among researchers and general public.

Studies of growth retardation in children with ADHD treated with CS in clinical and epidemiological studies report equivocal results. Some studies report growth retardation with catch-up of growth during drug-holidays or after ceasing treatment [14–17]; another study reports initial growth retardation with catch-up during CS treatment [18]; and yet another study found initial growth retardation with attenuation of the decreased growth velocity over time [19]. Of greater concern is, however, reports of growth retardation during CS treatment *without* later catch-up [20–23]. Finally, growth retardation in children with ADHD has also been reported to be independent of CS treatment, which has inspired the so-called maturation lag hypothesis [24].

It has been proposed that the growth retardation effect of CS treatment is dose-dependent [20, 23, 25–28] and may be limited to a certain subset of ADHD children [29, 30]. Conversely, other studies found no evidence to support such growth retardation or have questioned the clinical relevance hereof based on findings of normal growth parameters in adults who were treated with CS during childhood [17, 25, 27, 31–34]. A meta-analysis of 20 longitudinal studies concluded that height and weight were reduced compared to expected measures, but also that this effect attenuated over time [13].

Consensus holds that CS treatment is associated with initial growth retardation, but the implications of CS treatment for final height and stature remain unclear [6, 13, 19, 28, 30], among others because of methodological limitations in the above-mentioned studies pertaining to issues like different dose regimes, short follow-up and compliance problems. A further lack in the extant literature on this issue is the absence of studies of patients in continuous CS treatment from childhood through puberty into adulthood.

The dual aims of this study are, first, to determine the long-term effect of CS medication on linear growth and body weight in patients with ADHD; and, second, we aim to identify subgroups susceptible to increased risk of growth retardation.

In the present study of 410 patients treated with CS for an average of 6 years (range 0.9–16.1 years), we formulated five hypotheses: (1) Patients would experience an initial reduction in weight and halt in height; (2) Patients would catch up on growth parameters after 2–3 years of treatment; (3) There would be no gender differences as to hypotheses 1 and 2; (4) The following subgroups would be particularly susceptible to growth retardation: patients starting at a low age, patients with low weight prior to treatment, patients with autism or mental retardation, and patients with initial weight loss; (5)The growth retardation effect of CS treatment would be dose-dependent.

## Methods

The characteristics of the population, details regarding the procedures at the ADHD clinic, and the results of the annual growth measurements can be seen in Tables 1 and 2; the details have previously been published [35].

**Table 1:** Demographic data

	Male	Female	All
Gender	368	42	410
	90 %	10 %	100 %

	No	Yes	
Comorbidity	94	316	
	23 %	77 %	
Autism	373	37	
	91 %	9 %	
IQ below 90	369	41	
	90 %	10 %	
Tics/tourette	389	21	
	95 %	5 %	
CD/ODD	346	64	
	84 %	16 %	
Emotional disorder	390	20	
	95 %	5 %	
Learning disorder	185	225	
	45 %	55 %	
Disorder of social functioning	380	30	
	93 %	7 %	
Substance abuse	406	4	
	99 %	1 %	
Miscellaneous	390	20	
	95 %	5 %	
Age at start	Mean	Sd	
	9.2	2.4

**Table 2:** Distribution of number of weight and height measurements on 410 children

	Weight				Height			
	Number of measurements	No of subjects	% of all subjects (410)	Meas. per subject (range)	Number of measurements	No of subjects	% of all subjects (410)	Meas. per subject (range)
	0	6	1		0	7	2	
	1	14	3		1	14	3	
	2	27	7		2	33	8	
	3	55	13		3	58	14	
	4	65	16		4	68	17	
	5	69	17		5	73	18	
	6	56	14		6	58	14	
	7	43	10		7	35	9	
	8	27	7		8	30	7	
	9	17	4		9	20	5	
	10	15	4		10	8	2	
	11+	16	4		11+	6	1	
All periods	2209	404	99	(1; 24)	2056	403	98	(1; 15)
Period (month)								
−12 to −1 (before start)	322	293	71	(1; 4)	305	290	71	(1; 3)
0–11	328	212	52	(1; 9)	241	184	45	(1; 4)
12–47	863	356	87	(1; 11)	835	348	85	(1; 12)
48–71	393	228	56	(1; 12)	379	227	55	(1; 4)
72+	303	135	33	(1; 7)	296	134	33	(1; s6)

### Study design

The present study is a naturalistic observational study [36] of 410 participants with a diagnosis of ADHD or ADD treated with CS. Data on these patients were gathered at multiple, consecutive visits at the ADHD clinic at Aarhus University Hospital, Centre for Child and Adolescent Psychiatry, Denmark.

In the present study, it was not possible to differentiate between different types of CS with regards to substance (methylphenidate or dextroamphetamine) or with regards to short- vs. long-acting CS because patients changed medications several times in the course of the study.

### ADHD clinic procedure

Since 1998, the clinic has monitored patients aged 7–21 diagnosed with ADHD or ADD treated with CS. All patients attend the clinic at least annually (patients below the age of 18 are always accompanied by a primary caregiver). Two members of the medical staff are present at all visits. A consultant in child and adolescent psychiatry and a specialised nurse are always present in visits involving complex cases with severe comorbidity and/or medication besides CS; cases who present many adverse effects or side effects of medication; and cases with severe behavioural, educational or malfunction problems. Visits involving less complex cases were staffed by two specialised nurses who could consult the psychiatrist if any questions arouse. These cases were then reviewed by the child and adolescent psychiatrist at weekly clinical conferences.

### Assessment of main diagnosis at initial assessment

The patients were primarily diagnosed at a cross-disciplinary conference after having been assessed through a review encompassing their full medical and psychiatric history; observation at school and leisure activities; physiological examination and clinical assessment including neurological examination and motor function tests; psychological examination (at least WISC); and a report form (most often ADHD-RS) completed by parents, school and leisure time teachers describing the patients’ ADHD symptoms and symptom severity. For patients diagnosed in their teens, observations and motor function tests were often replaced by an interview of the patient.

### Assessment of comorbidity

Depending on age at baseline, all the patients were assessed for psychiatric comorbidity by using Kiddie-SADS, DAWBA or another structural clinical interview. The choice of diagnostic tool and the different tools used in the study reflect the development of diagnostic instruments during the study period. Patients included more than 10 years ago were more likely to have been assessed by a local structured clinical interview on the basis of diagnostic criteria for child and adolescent psychiatric disorders, whereas children included during the past 7–8 years were more likely to have been assessed by Kiddie-SADS or DAWBA. A comorbid diagnosis was given either after the primary assessment concomitantly with the main diagnosis or later after a new cross-disciplinary clinical assessment had been performed in which an MD participated. The latter assessment was combined with semi-structured interviews or report forms and a psychological examination when necessary. In cases where the clinical assessment raised suspicion of a diagnosis of pervasive developmental disorder, an ADOS and/or ADI was performed [37].

### Assessment of growth measures (anthropometric assessment)

Height was measured in cm without shoes from the sole of the foot (the floor) to the vertex of the skull, and weight was assessed in kilos with the subject wearing indoor clothing without shoes.

We used the most recent Scandinavian growth tables [38, 39] to convert weight and height measures into age- and sex-adjusted z-scores, i.e. the difference between the observed value and the excepted value divided by the standard deviation found in the growth tables for the given age and sex.

Our calculations of z-scores are based on Swedish population norms. This approach is in line with recent Danish paediatric recommendations which argue that the Danish population is comparable to the Swedish regarding growth data [38, 39]. The Swedish growth curves from 2002 are based on growth data from a retrospective longitudinal study of 3650 full-term healthy children born between 1973 and 1975 who were all in the 10th grade at school in the town of Gothenburg, Sweden. The children’s final height was measured at the time of the study, and previous height measures were obtained retrospectively by examining the children’s health records. The cohort was socioeconomically representative for Swedish children. Children born before the 37th week and children with a chronic disease were excluded. The data are representative for Danish children according to the most recent weight and height curves available.

### Database

In collaboration with two specialists in child and adolescent psychiatry and a statistician, a database was compiled consisting of (1) individual factors: date of birth, gender, date of medication start, comorbidity and co-medication; (2) changes in CS: date of any change, type of medication (Ritalin^®^, Ritalin Uno^®^, Concerta^®^, dexamphetamine and Strattera^®^) and dosage (total daily dose and number of doses) and the reason for change; (3) visits at the clinic: date, weight, height, pulse, blood pressure, effect and adverse effects of medication, ADHD-RS scores, SDQ scores, diagnosis and C-GAS scores; (4) all height and weight measurements registered since start of medication; (5) treatment status at the end of the survey or the end of the clinical visits.

### Statistics/data analysis

In order to describe the possible change over time in the z-score for weight and height, we divided the time into five periods: the year before treatment start (baseline period) (−12 to −1 month), the first year after start (0–11 months), year 2–14 (12–47 months), year 5–6 (48–71 months), and more than 6 years after start (72+ months).

In order to describe the associations between average dose per kg and weight and height z-scores, we calculated the difference in daily weight and height scores between any two visits. For each date we set the dose to be the true dose, i.e. the latest prescribed dose, while the weight and z-scores were found by interpolation of the latest and the next measured values. From this, we could calculate the dose per kg for each day while taking into account the variation in each child’s dose and weight during the study period. Based on these expanded data, we calculated the average dose per kg, height and weight z-scores for each subject for each time interval.

The analyses of weight and height were based on the observed measurements and included data for all subjects who were measured at least once since 1 year before the start of treatment. The z-scores were analysed by repeated measurements models with random subject levels, and the correlation between two observations within subject decreasing with the time span between the measurements (Gaussian autocorrelation). This model specification implies that we can analyse data for all subjects even though some subjects only contributed with one or a few observations. First, we analysed the general development over the five time intervals. Second, we analysed whether the development in growth parameters after start of CS treatment was influenced by age at treatment start, sex, autism, IQ or emotional disorder.

We tested the following three models: (1) parallel curves, (2) parallel curves after treatment start: and if the first two models could be accepted (3) no differences between groups.

Data management and statistical analyses were made in Stata 12.0 and SAS 9.2 [40, 41]; estimates are presented with 95 % confidence intervals (CIs); and p values below 0.05 are considered statistically significant.

## Results

### Demographics

In total, 417 participants were identified representing the entire population of patients with ADHD assessed at the clinic during the study period. A total of 410 medical charts were reviewed. Seven charts were unavailable.

A total of 368 of the probands were male (90 %), 136 (77 %) had one or more comorbidities: 37 autism, 21 Tourette’s syndrome (TS) or tics, 64 conduct disorder or ODD, 20 emotional disorders, 225 learning disorders and 4 substance abuse (Table 1). In 41 subjects, the IQ total score was below 90. Medication started in 74 (18 %) at the age of 3–6 years, in 204 (50 %) at 7–9 years, in 100 (24 %) at 10–12 years, and in 32 (8 %) at 13 years or older. The mean age at medication start was 9.2 years (range 3.3–17.6). The mean observation time was 6.0 years (range 0.9–16.1).

The number of measurements of weight and height is seen in Table 2.

### Growth measures over time

**Fig. 1:**
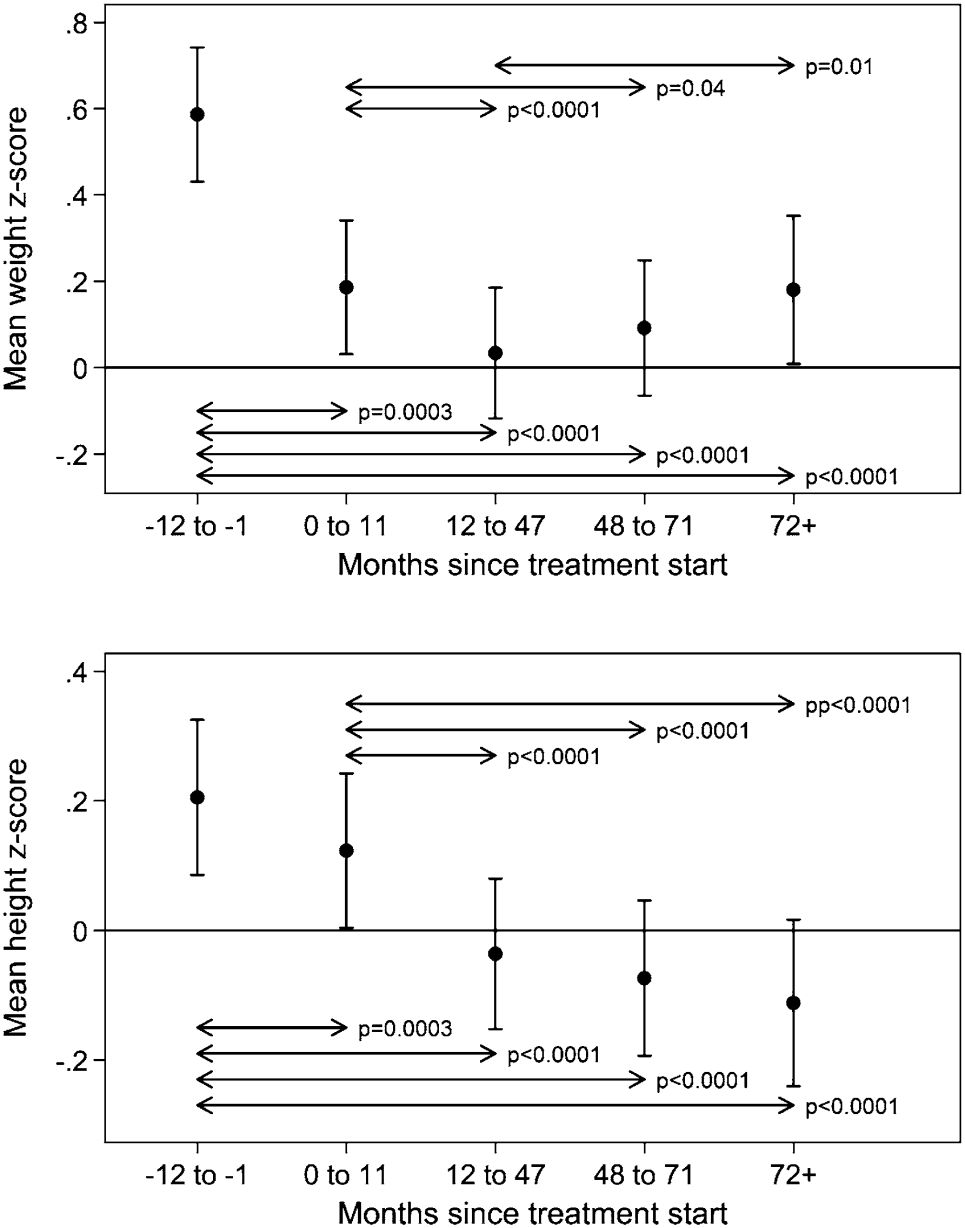
Weight and height z-scores at baseline and at 0–11 months, 12–47 months, 48–71 months and 72+ months after treatment start for the entire population. The p values for statistically significant differences between time groups are marked. The *bars* indicate 95 % confidence intervals

The average z-scores for weight and height at baseline and at the different time intervals are illustrated in Fig. 1. At baseline, z-scores were significantly above the normative data for weight [M = 0.59, 95 % CI (0.43–0.74), p < 0.0001] and height (M = 0.21, p = 0.001), which indicates a larger than expected relative weight and height.

We observed a significant reduction in z-score from baseline to any time interval investigated (p < 0.0001); the largest decrease occurred in the interval from baseline to 12–47 months [M = −0.55 CI (−0.63; −0.48)], see Fig. 1. From 0–11 to 12–47 months and to 48–71 months, a significant difference in z-scores was observed [M = −0.15; CI (−0.21; −0.09); p < 0.0001] and [M = −0.09 (−0.18; 0.00); p = 0.04], respectively. From 12–47 to 48–71 months, z-scores plateaued [0.06; (−0.01; 0.13); p = 0.11]; but from 12–47 to 72+ months, z-score increased [M = 0.15; (0.04; 0.26) p = 0.01]. The latter data include a similar z-score from 48–71 months to 72+ months [M = 0.09; (0.00; 0.18) p = 0.06].

Height z-scores decreased from baseline to any time interval investigated (p < 0.003 to p < −0.001). The total absolute reduction was 0.32, (Fig. 1). From 0–11 months to the following time intervals, a significant decrease was also found (p < 0.0001); the total absolute difference was 0.24.

Z-scores did not exhibit a time-dependent rebound effect in the latter time periods.

From 12–47 months and onwards, the decrease in z-score was constant from time point to time point: 12–47 months vs. 48–71 months (−0.04; p = 0.15); 12–47 months vs. 72+ months (−0.07: p = 0.05); 48–71 months vs. 72+ months (−0.03; p = 0.24); these decreases did not reach clinical significance and they indicate a plateauing of the z-score.

### Moderators of growth

#### Gender

**Fig. 2:**
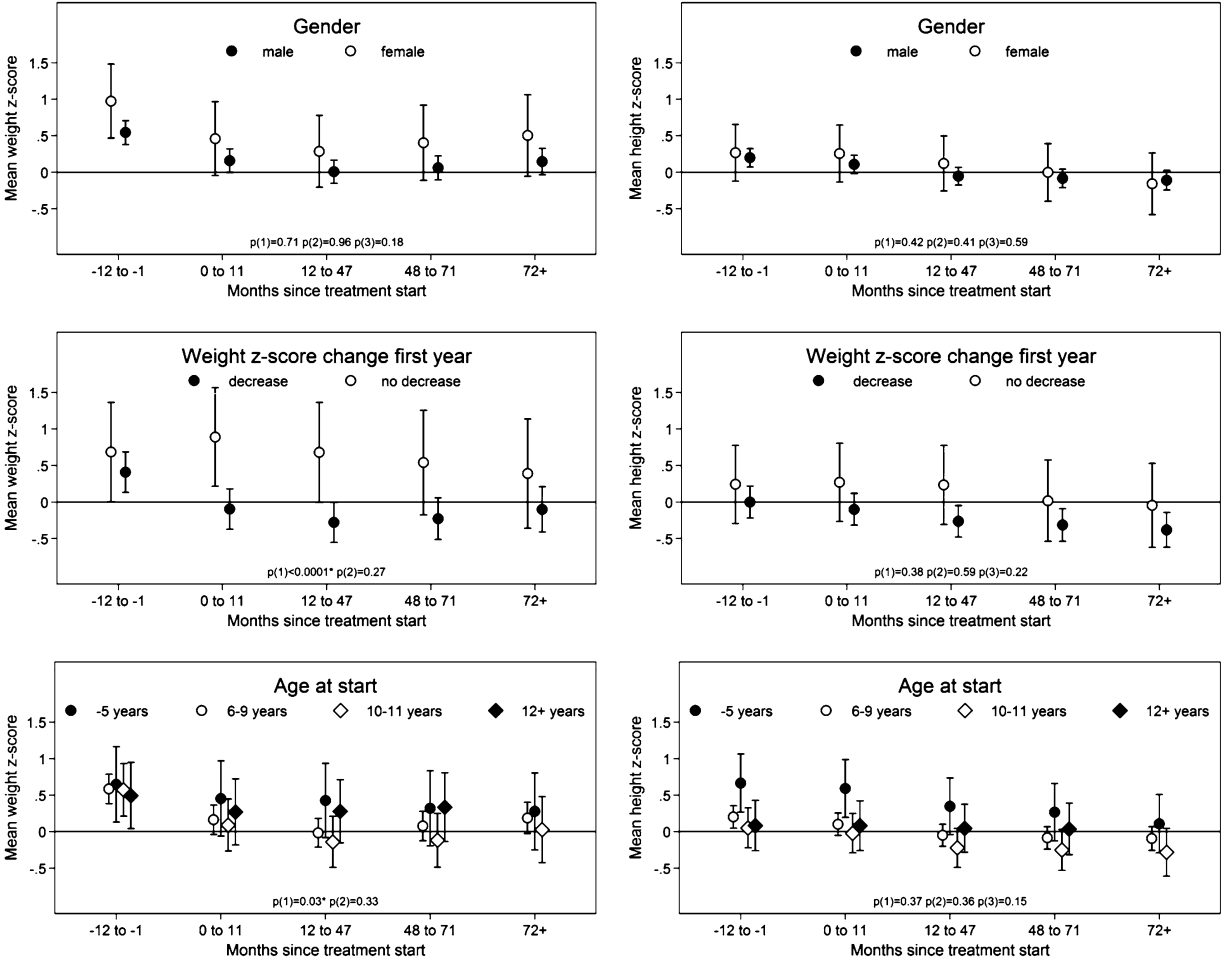
Weight and height z scores at baseline and in 0–11 months, 12–47 months, 48–71 months and 72+ months after treatment start according to gender, age at treatment start and change in weight z score within the first year of treatment. The bars indicate 95 % confidence intervals. p(1): p value for test for the entire course of curves among groups (i.e. are the curves parallel?). p(2): p value for test for the course of the curves after 0–11 months among groups (i.e. are the courses of the curves the same after 0–11 months?). p(3): p value for test for no group effect given the curves are parallel. If p(1) or p(2) reaches significance p(3) is omitted. The *bars* indicate 95 % confidence intervals

Means of z-scores were identical in the two groups of boys and girls (weight p = 0.18, and height p = 0.59) (Fig. 2). Gender was not associated with the course of the curves for z-weight throughout the entire time period (p = 0.71) nor from 0–11 months onwards (p = 0.96). Neither was this the case for z-height (p = 0.42 for the entire treatment time and p = 0.41 from 0–11 months onwards).

In order to analyse whether a decrease in weight within the first year of CS treatment was a predictor for a permanent weight loss, we identified a group having a significant decrease in weight z-score during the first year of treatment (group 1, N = 137) and compared this group with subjects without such a decrease (group 2, N = 23), Fig. 2.

At baseline, z-scores *for weight* were significantly above the normative data for both groups [M = 0.41; CI (0.13; 0.69); p = 0.0043 for group 1 and M = 0.69; CI (0.006; 1.364) p = 0.049 for group 2]. Z-scores for weight over time for the 2 groups differed significantly over the entire treatment period (p < 0.0001), but from 0–11 months onwards the curves were similar (p = 0.27).

Altogether, we saw a decrease in weight from baseline of 0.30; this reduction ended at the level of z-score, i.e. within the normative range (M = 0.39, p = 0.31). In group 1, the z-score for weight continued to decrease, but to a lesser degree than the overall z-score. This continued until the 12–47 month period (M = −0.18), resulting in a total difference of 0.69 from baseline. From then on, weight z-scores attenuated slightly and reached a z-score level for weight within the normative range (M = −0.1; p = 0.53). For group 1, the lowest z-score was significantly below the normative range (M = −0.28; p = 0.0498), whereas the lowest z-score for group 2 was within the normative range (M = 0.39; p = 0.31).

The two groups did not differ regarding z-score *for height* (p = 0.22). At baseline, both groups had heights comparable to normative data (group 1 with a height z-score = M = 0; p = 0.99, group 2 with a height z-score = M = 0.24; p = 0.38).

The two groups did not differ significantly over the entire period (p = 0.38) or from the 0–11-month period and onwards (p = 0.84). Noteworthy is, though, that from the 12–47-month period and onwards, group 1 had z-scores for height below normative data. Group 2 z-scores remained comparable to those of the normative data over the entire observation period despite the fact that they experienced a decline.

Analysis of CS doses revealed that subjects with a weight z-score decrease in the first treatment year received significantly higher doses than the rest of the study population, not only in the first treatment year, but also in the following time intervals.

#### Age at start of treatment with medication

The possible impact the age at start of medication may have on z-scores is shown in Fig. 2. We have shown the z-scores over time for the following age groups: up to 5 years, 6–9 years, 10–11 years and 12+ years. We found that *weight z*-*scores* were significantly different over the entire period, but similar after the 0–11-month period and onwards. The size of the change in z-score during the first year of treatment was not related to age. Thus, the largest negative changes in z-scores were found in the 6–9-year-old and 10–11-year-old starters. The up-to-5-year-old starters and the 12+ year–old starters thus proved to have a relatively smaller decline in z-score during the first treatment year.

*Height z*-*scores* were similar over the entire period (M = 0.37) and from 0–11 months onwards (p = 0.36).

Subjects starting medication below the age of 6 generally tended to be taller than those starting medication later—this was not statistically significant, though. We found that patients starting medication below the age of 6 showed a tendency towards higher dosages throughout the entire treatment period. The difference in CS doses became significant in the 12–47-month period (M = 0.80 vs. M = 0.96, p = 0.007), in he 48–71-month period (0.76 vs. 1.00 mg/kg, p < 0.001) and in the 72+ time period (0.70 vs. 1.05 mg/kg, p < 0.0004).

#### Z-score prior to treatment

**Fig. 3:**
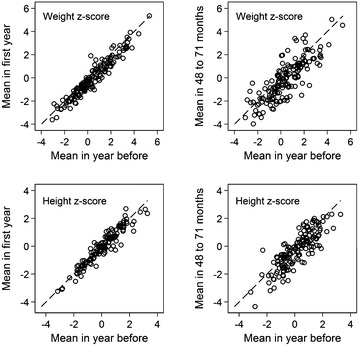
Weight and height z-scores at baseline plotted against weight and height z-scores at 0–11 months and 48–71 months respectively

Figure 3 illustrates z-score for weight and height in the year before treatment in relation to z-scores in the first treatment year and 4–6 years after treatment was initiation. The figure does not indicate differences in the susceptibility to changes in z-scores in accordance with lower or higher z-scores at baseline.

#### Dose

For *weight*, there was a dosage effect on the magnitude of change in z-scores—the larger the dose, the greater fall in z-score in all time periods. Furthermore, the change in z-score for the ≥1.5 mg/kg group continued to increase also in the 72+ month period; at this time reflecting an attenuation in the two other dosage groups.

For *height* there was no clear dosage effect in the 0–11 month period. For the rest of the time periods, the dosage effect was clear: the higher the dose, the larger the fall in z-score from baseline. The 48–71-month periods stood out as exceptions with a fall in z-scores for the 0.5–1.4 mg/kg and the ≥1.5 mg/kg groups of similar magnitude. In the 0–0.4 mg/kg group, an attenuation of the change in z-score was observed at 72+ months at which time the other two dosage groups revealed a continued fall from baseline.

Table 3 illustrates the change in weight and height z-score from baseline for the different time periods in relation to the dosage (mg/kg) given in the time period before, i.e. change in z-scores in the 12–47-month period was related to dosage in the 0–11-month period.

**Table 3:** Weight and height z-scores according to month since start of treatment and average CS dose in period

Average dose in previous period	Period (months from start)			
	0–11	12–47	48–71	72+
Weight z-score change since baseline				
0–0.4 mg/kg				
Number of persons	332	52	31	21
Average (SD)	−0.24 (0.32)	−0.26 (0.70)	−0.15 (1.04)	−0.32 (0.90)
0.5–1.4 mg/kg				
Number of persons		271	165	83
Average (SD)		−0.51 (0.64)	−0.48 (0.89)	−0.25 (1.00)
1.5+ mg/kg				
Number of persons		6	4	1
Average (SD)		−0.61 (0.21)	−0.93 (0.13)	−1.47 (0.00)
Height z-score change since baseline				
0–0.4 mg/kg				
Number of persons	331	51	30	21
Average (SD)	−0.08 (0.20)	−0.15 (0.41)	−0.16 (0.71)	−0.44 (0.60)
0.5–1.4 mg/kg				
Number of persons		271	165	83
Average (SD)		−0.28 (0.44)	−0.47 (0.64)	−0.51 (0.75)
1.5+ mg/kg				
Number of persons		6	47	1
Average (SD)		−0.43 (0.25)	−0.89 (0.45)	−0.12 (0.00)

The change since baseline was negatively associated with the dose in the previous period both for weight and height. Thus, the mean z-score for weight decreased by 0.52 (95 % CI 0.33; 0.70) per mg/kg dose, while the mean z-score for height decreased by 0.33 (95 % CI 0.19; 0.47) per mg/kg compared with the previous period.

#### Comorbid autism

**Fig. 4:**
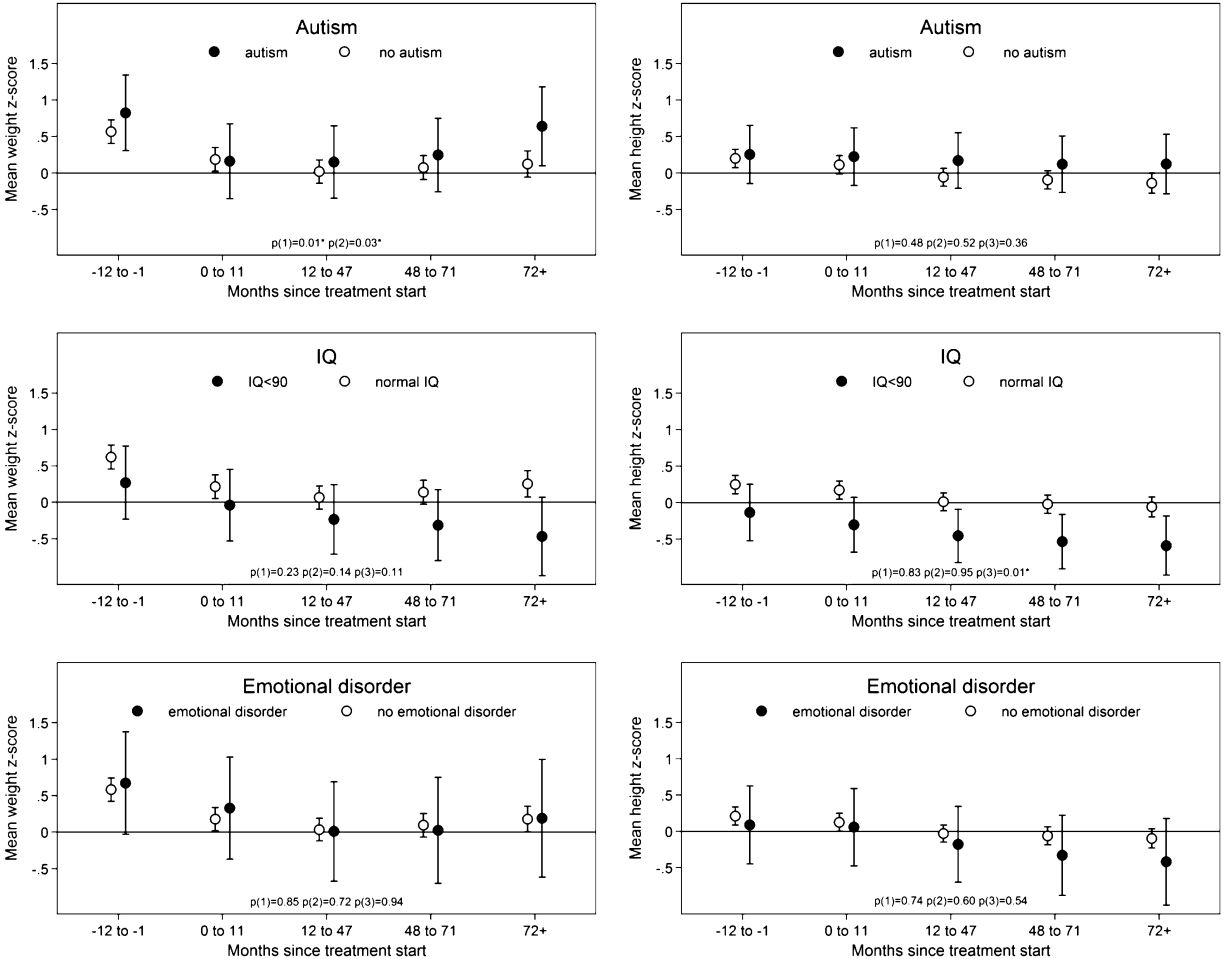
Weight and height z scores at baseline and at 0–11 months, 12–47 months, 48–71 months and 72+ months after treatment start for subjects ± autism subjects, ± IQ below 90 subjects and ± emotional disorder subjects. p(1): p value for test for the entire course of curves among groups (i.e. are the curves parallel?). p(2): p value for test for the course of the curves after 0–11 months among groups (i.e. are the courses of the curves the same after 0–11 months?). p(3): p value for test for no group effect given the curves are parallel. If p(1) or p(2) reaches significance p(3) is omitted. Weight and height z scores at baseline and at 0–11 months, 12–47 months, 48–71 months and 72+ months after treatment start for subjects below or at/over 6 years of age at treatment start. The *bars* indicate 95 % confidence intervals

At baseline, subjects with and without autism both had weight z-scores significantly above the normative data (M = 0.82; p = 0.002; 0.57; p < 0.001, respectively) Fig. 4.

Subjects with autism demonstrated significantly different changes in their z-scores for weight over the entire time period (p = 0.01) and from 0–11 months onwards (p = 0.03) compared with non-autistic subjects. They had a steeper decline in weight z-score from baseline to 0-11 months (M = 0.66 vs. M = 0.38), a smaller decrease from 0–11 to 12–47 months (M = 0.01 vs. M = 0.17) and a greater increase in z-score from 12–47 months and onwards compared with the remaining subjects. In the 72+ time group, the autistic subjects again reached a z-score significantly greater above the normative data (M = 0.64; p = 0.02), whereas the z-score of non-autistic subjects was insignificant compared with the normative data (M = 0.12; p = 0.18) despite their baseline z-scores.

Height z-scores were similar in autistic and non-autistic subjects (p = 0.36). Having an autism diagnosis did not affect the courses of the curves for z-height over the entire time period (p = 0.48) or from 0–11 months onwards (p = 0.52).

#### IQ below 90

No effect of low IQ was seen in regards to z-scores for weight over the entire time period (p = 0.23) or from 0–11 months onwards (p = 0.14), although there was a trend towards a continued decrease beyond 12–47 months for low IQ subjects.

Subjects with low IQ generally had a lower height than the other children (M = −0.47; p = 0.01). At baseline, z-scores for height were above expected values for subjects with a normal IQ (M = 0.25; p < 0.001), whereas subjects with low IQ had z-scores comparable to normative data (M = −0.14; p = 0.49) There was no effect of low IQ on z-scores for height over the entire time period (p = 0.83) or from 0–11 months onwards (p = 0.95), but subjects with low IQ had reached a height z-score lower than normative data (M = −0.6; p = 0.0045) at 72+ months, whereas normal IQ subjects had height scores comparable to the normative data (M = −0.1; p = 0.39).

Dose analysis revealed that patients with low IQ had similar doses as the rest of the study population in the 0–11-month and the 12–47-month periods, but significantly higher dosages in the 48–71-month period (M = 0.76 vs. 0.99 mg/kg for normal and low IQ subjects respectively, p = 0.002) and the 72+ month period (M = 0.72 vs. 1.17 for normal and low IQ subjects respectively, p = 0.001).

#### Emotional and behavioural disorder

Subjects with an emotional disorder (including emotional disorders in childhood and depression according to the ICD-10 classification did not differ from others with regards to z-scores for weight (p = 0.94) or height (p = 0.54). We observed no effect of emotional disorder on z-scores for weight or height over the entire time period (p = 0.85 and p = 0.74, respectively) or from 0–11 months onwards (p = 0.72 and p = 0.60, respectively). Subjects with ODD or CD did not differ from others with regards to z-scores over time for weight (p = 0.68) or for height (p = 0.69), and the general level of z-scores did not differ either for weight (p = 0.92) or height (p = 0.61).

## Discussion

The present study of the long-term effects of central stimulants on growth parameters in patients with ADHD and ADD is unique with regards to the number of patients included, the length of the observation period and the number of regular assessments. The main findings of our study are that (1) CS treatment of patients with ADHD led to a relative decrease of body weight and height, (2) the relative decrease of body weight stagnated after 12–47 months of CS treatment as did the halt in height growth; even after 72 months of CS treatment the patients had not returned to their baseline body weight and height values, and (3) doses and z-score decreases were negatively associated.

Subgroup analyses revealed that patients with a relative weight loss within the first 12 months of treatment suffered a larger and longer-lasting reduction in relative weight; and patients with concomitant ASD exhibited a faster and more profound relative weight loss.

The decrease in z-score for weight reported here is in line with numerous previous studies. The onset of catch-up in weight z-score from 12–47 months occurred later than in many other studies, and the z-score remained below baseline for patients observed at 72 months or later. This contrasts with the findings of Biedermann et al. [34] who recorded no effect of CS on adult body weight in a 10-year prospective study. Inversely, our findings confirm the conclusions of the MTA study, albeit it analyses the effects of CS treatment over a longer period. Whether the patients’ relative weight loss observed at 72 months or more was clinically relevant may be questioned because the patients’ relative body weight was above the normative level at this time point. The patients weight and/or height were above normative levels prior to medication start as also observed for this patient group in several other studies [12, 19, 21, 25, 27, 42]; and this fact argues against the maturational lag hypothesis [24].

Our finding that the decline in z-height growth over time plateaued from 12–47 months without reaching baseline, but remained within the expected range for age, support the existing literature [17, 25, 27, 33, 34] that has questioned the clinical relevance of reduced growth rates by finding normal growth parameters in adults treated with stimulants in childhood. However, there may be subgroups for whom initial weight loss and attenuation of height velocity may have an impact [30].

Comparison of height and weight with normative data in the absence of standardised growth tables for patients with ADHD may cause conclusions about the effect of medication on the observed growth parameters to be overestimated if ADHD patients have differential growth patterns unrelated to their medication status [24].

We found no gender effect on growth parameters, which is in line with the literature.

### Decrease in z-score in the first year

We found that patients who suffered no weight loss during their first year of treatment lost weight later. For patients with a weight loss in the first treatment year, the total weight loss was more severe and their lowest z-score was below normative data. However, only 162 persons were weighed both at baseline and in the first treatment year even though 212 subjects were weighed at baseline. Thus 52 patients who were weighed at baseline were not weighed during the first treatment year, and we therefore do not know whether they had a change in z-score. Assuming that the patients who were not weighed were likely to have minor weight problems, we may argue that weight loss in the first year of CS treatment is a predictor for weight loss over a longer period of time *and* a greater weight loss in general and therefore for weight deficits over time.

The group with a significant weight loss in the first treatment year received a significantly higher CS dose than the group without weight loss in the first treatment year. Although no causality can be proved, this difference in dosage may be an explanation.

### Age at start of stimulant treatment

We expected that CS treatment from an early age would have a larger impact on growth parameters than treatment start at an older age; but, in fact, we saw the opposite. This finding could not be explained by a more cautious titration of dosage among these young starters as they were treated with high dosages [35]. This contrasts with the literature documenting greater susceptibility to adverse effects among preschool children [12]. We found no impact of differing age at start regarding z-score over time.

Our data revealed no association between low or high weight and/or height z-scores prior to treatment and differing susceptibility to weight or height deficits over time. This is not in line with the PATS study, which concluded that the greatest weight loss may be found in patients overweight prior to treatment [12].

### Dosage

The impact on z-score correlated with dosage. The dosage effect on z-score was clear even after several years of treatment and could also be seen at low dosages. In line with earlier studies, growth retardation was seen at all dosages, but not in all subjects [20]. Thus, we conclude that other individual factors have an impact on z-score changes. An important bias here is that, overall, only few subjects were treated with high dosages, which decreases the statistical accuracy of this calculation.

### Comorbid autism

Patients with autism experienced a larger decrease in weight z-score from baseline and until the 0–11-month period. The mechanisms lying at the root of the increased effect on weight in subjects with ASD remain to be investigated. In a previous paper [35], we found that autistic subjects did not differ from the other patients regarding CS dosage which rules out dosing differences as an explanation. Their weight z-scores attenuated earlier, and at 72+ months their weight z-scores reached a level significantly above normative data, and CS dosage therefore had almost no long-term impact on weight. Co-medication with orexigenic antipsychotics frequently used in this patient group [43] was not more frequent among autistic subjects than among non-autistic subjects which rules out orexigenic antipsychotics as an explanation. Selection bias may be an explanation if autistic subjects with growth retardation exited the clinic more frequently than autistic subjects without problems of growth retardation.

Despite the impact of autism on z-scores for weight, no impact of autism on the z-scores for height was revealed.

### IQ

Patients with an IQ below 90 had a tendency to experience a continued decline in weight z-score, although the decline fell short of statistical significance.

In a previous paper, we concluded that patients with an IQ below 90 received significantly higher average CS doses, had significantly larger dose increases over time and were being treated with high doses (>1.5 mg/kg) significantly longer and with low doses (<1 mg/kg) significantly less than others. These differences in CS dosage may explain the differences in weight z-scores [35].

Patients with a low IQ generally had lower height z-scores than patients with a normal IQ, but we found no differences regarding the magnitude of the decrease in height z-score over time. Of clinical relevance, though, is the finding that subjects with a low IQ at 72+ months had a height z-score below the normative level even though they had a z-score corresponding to their norm at baseline. Inversely, normal-IQ subjects experienced a change from a larger than expected height z-score to a z-score corresponding to the expected level. This indicates that CS treatment has a more serious impact on the height of low-IQ subjects than on the height of normal-IQ subjects.

### Emotional disorders

Biedermann proposed that major depressive disorder combined with ADHD influence the z-scores for weight for both genders, but in opposite directions with boys exhibiting lower z-scores and girls higher than expected z-scores [34]. In our study, we recorded no effect of emotional disorders on z-scores; but, on the other hand, we did not specifically perform statistical analyses on the group of girls because the number of girls was low. Moreover, the proportion of subjects with emotional disorders was low, probably because only diagnoses present at the end of observation period were included. Another explanation is that emotional disorders may have been overlooked because their symptoms were considered part of the ADHD entity [35].

### Strengths and limitations

Our study has several methodological shortcomings, the most important of which relate to its retrospective design. Patients have entered and exited the clinic at different stages of treatment and for varying reasons which creates problems of selection and information bias. The lack of control group is also a significant limitation in the present study. However, a control group is often not an option in clinical samples due to lack of clinical equipoise of stimulants.

The observation time was long for most patients but differed due to dropout of medication, dropout of clinic or, merely, treatment start just before the study period ended. Since we do not know whether patients with the longest follow-up differ from other patients with regards to growth parameters, the impact of this bias is unknown. If subjects contributing to the 72+ month period had lower z-scores before treatment than subjects who did not contribute to this period, the effect of CS treatment on the z-score may be overestimated. If subjects with growth problems cease medication earlier, the effect of CS on growth may be underestimated. Our previous paper concluded that those remaining in the clinic receive larger doses than those ending medication or those who are transferred to their GP [35]. Another issue of concern is the varying number of growth measurements between subjects and over time. Some patients were weighed more frequently than others, and this is a bias if those who were weighed more often differed from those who were not. Compliance problems, interruptions in treatment and dosage changes are likewise evident issues of concern in this study design, but these disparities reflect the clinical reality when treating ADHD patients.

No pubertal staging was stated in the medical charts, and heterogeneity of puberty and growth speed may therefore influence our results [26].

Co-medication was used at some point. Insufficient data regarding dosage, timing and duration of this treatment excluded this parameter in our analysis. I it is well known that other psychopharmacological medications also influence appetite, caloric intake and growth parameters; and any administration of such medications may bias our conclusions.

This study also has a number of strengths and identifies important issues that should be considered when treating real-life patients with ADHD. The strengths of the present study include a large population of patients, long-time follow-up of patients in continuous stimulant treatment, regular assessments, precise and systematic registration of stimulant dose over time, clinical subgroups which can be compared (comorbidity, age, gender, time of treatment), and patients were stimulant-naïve at baseline before entering study registration.

## Conclusions

The present study contributes with valuable information relevant both to child and adolescent psychiatrists, adult psychiatrists and paediatricians. Our study identifies important issues relevant when compiling clinical recommendations for CS treatment of patients with ADHD.

Our study shows that growth measurements need to be continuously taken into account when treating children and adolescents with stimulants.

A need remains for further clinical studies of the impact of growth retardation on final adult height and weight. More specifically, further identification of susceptible subgroups is needed, the effect of stimulant dosage should be elucidated, and studies unravelling the anorexic and metabolic mechanisms of stimulants are warranted.

## Highlights

- We found a fall in relative weight and in height growth for patients in CS treatment; there was an attenuation in the decrease after 12–47 months of treatment, but baseline values had not been reached at 72+ months.
- Changes in weight and height parameters were dose-related.
- Patients with decrease in relative weight within the first 12 months experienced a more profound relative weight loss.
- Patients with weight loss in the first year experienced a more serious relative height deficit.
- Children with comorbid autism had a steeper decrease in relative weight initially but regained a weight z-score above normative data.
- Correlations with weight and height in the subgroup with low IQ was probably dose-related.
- We did not find that early start of treatment led to a higher decrease in z-scores on weight or height.
- We found no relation to weight or height regarding gender, comorbid ODD/CD or emotional disorders.
